# Association of Body Metrics and Ocular Diseases

**DOI:** 10.3390/jcm14165835

**Published:** 2025-08-18

**Authors:** Hae-Nah Gwon, Hye-Jin Son, Young-Joo Shin

**Affiliations:** 1 Department of Ophthalmology, Hallym University Medical Center, Hallym University College of Medicine, Seoul 07441, Republic of Korea; ghn9107@naver.com (H.-N.G.); shj3534@hallym.ac.kr (H.-J.S.); 2 Hallym BioEyeTech Research Center, Hallym University College of Medicine, Seoul 07442, Republic of Korea

**Keywords:** body metrics, ocular diseases, cataracts

## Abstract

**Background/Objectives**: The relationship between systemic health and ocular diseases is well-documented, with various body metrics potentially playing significant roles in the pathogenesis of cataracts, glaucoma, and age-related macular degeneration (AMD). However, comprehensive studies linking these metrics with ocular health are sparse. This study aims to explore the associations between height, weight, waist circumference, and BMI with the prevalence and current status of cataracts, glaucoma, and AMD in a large cohort. **Methods**: We used data from Korean National Health and Nutrition Survey (KNHANES 2015–2021), a national, cross-sectional health examination and survey, for which representative data on the health, nutritional status, and physical activities of the Korean general population are collected by the Korea Centers for Disease Control and Prevention (KCDC). We compared height, weight, waist circumference, and BMI among patients with diagnosed and current cataracts, glaucoma, and AMD versus those without these conditions. Statistical analyses included *t*-tests and Pearson correlation analyses to examine the relationships between body metrics and ocular diseases. **Results**: Our findings indicate that shorter height and lower weight are associated with diagnosed cataracts and glaucoma but not with their current status. A greater waist circumference was observed in patients with diagnosed cataracts, glaucoma, and AMD compared to controls, suggesting central obesity as a potential associated factor. No significant differences in BMI were found in patients with current ocular diseases. Additionally, certain body metrics were correlated with refractive errors and visual acuity, suggesting broader implications for ocular health. **Conclusions**: The study highlights significant associations between body metrics and the risk of developing cataracts, glaucoma, and AMD. AMD was found to be more closely related to systemic diseases, such as diabetes and hypertension, than to body metrics. These findings suggest that interventions targeting obesity and metabolic health could potentially reduce the risk or severity of these common ocular conditions. Further research is needed to confirm these relationships and explore underlying mechanisms.

## 1. Introduction

The relationship between body height and ocular diseases presents a complex and multifaceted landscape in ophthalmic research. Numerous studies have revealed intriguing associations between an individual’s stature and various aspects of ocular health, including structural characteristics of the eye and the risk of developing certain ophthalmological conditions [1,2]. The systemic effects of body composition on vascular health, metabolic processes, and inflammatory pathways may influence the pathogenesis of eye conditions [3,4]. Body metrics, such as height, weight, waist circumference, and body mass index (BMI), are not only indicators of general health but may also be associated with various ocular diseases, including cataracts, glaucoma, and age-related macular degeneration (AMD) [4]. Cataracts, the clouding of the lens, are primarily associated with aging but are also influenced by other risk factors including diabetes, smoking, and possibly body composition [5,6]. Glaucoma, a group of diseases that damage the optic nerve, has potential links to vascular health and intraocular pressure, which may be affected by obesity and other aspects of physical health [7,8]. AMD affects the retina and is the leading cause of vision loss among older adults, with risk factors including age, smoking, and possibly body size and cardiovascular health [9,10].

Despite the established links between these factors and ocular diseases, few studies have comprehensively explored how each body metric relates to the presence and severity of these conditions [8]. Moreover, the role of these body metrics in the management and prognosis of eye health remains underexplored. Thus, our study aims to fill these gaps by investigating the relationships between body metrics and the prevalence, as well as the current state, of cataracts, glaucoma, and AMD in a large study population. Through this approach, we hope to elucidate potential targets for intervention and prevention, contributing to the broader understanding of how systemic health impacts ocular diseases. Thus, we investigated the relationships between various body metrics—specifically height, weight, waist circumference, and body mass index (BMI)—and the prevalence and characteristics of major ocular diseases, such as cataracts, glaucoma, and AMD.

## 2. Materials and Methods

### 2.1. Ethics Statement

The Korean National Health and Nutrition Examination Survey (KNHANES) was approved by the Korean Centers for Disease Control and Prevention Institutional Review Board, and all participants provided written informed consent. This study adhered to the tenets of the Declaration of Helsinki.

### 2.2. Study Population

For this cross-sectional, population-based study, we used data from Korean National Health and Nutrition Survey 2017–2021, which is a series of cross-sectional surveys of nationally representative samples of the civilian Korean population aged 1 year and older that are conducted annually to assess the health and nutrition status of the South Korean population. To obtain representative samples, the KNHANES uses a stratified, multistage, cluster probability sampling design according to geographical area, age, and gender. For the health interview survey, a trained interviewer asked questions directly to individuals aged >19 years. This study included 34,372 adults (15,190 men and 19,182 women) aged >19 years who met the eligibility criteria and who completed a questionnaire regarding independent associated factors and underwent slit-lamp examinations.

### 2.3. Data Collection and Diagnostic Criteria

For accurate data collection, two questions, namely “Have you ever been diagnosed with cataracts by a doctor?” and “Do you have cataract, glaucoma and AMD?”, were used to assess self-reported information about cataracts, glaucoma and AMD. The possible responses to the question about a previous diagnosis were “No” or “Yes”. All participants underwent a corrected visual acuity test, with autorefraction fundus examination and intraocular pressure measurement. Data collected by the 2015–2021 KNHANES were analyzed in the present study [11]. Unmatched or no response (untested) were removed. The population was categorized based on age (young: <60 y, old: ≥60 y), height (short: <163 cm, tall: ≥163 cm), weight (light: <64.2 kg, heavy: ≥64.2 kg), waist circumference (small: <84 cm, great: ≥84 cm), and waist circumference-to-height ratio (low: <0.5, high: ≥0.5). Diabetes mellitus, hypertension, physical activity, and smoking were also adjusted as a potential associated factor.

### 2.4. Statistics

Statistical analyses were performed by using SPSS Version 27.0 (SPSS Inc., IBM Software, Portsmouth, UK). *p*-values < 0.05 were considered statistically significant. An independent *t*-test was used for analysis between two groups. The chi-square test was used for the comparison of discrete variables between groups. To estimate odds ratios (ORs) of dry eye and potential factors, we conducted logistic regression analyses by using the generalized linear model for a complex survey design. The ORs and 95% confidence intervals (CIs) were calculated in confounder adjustment for age, sex, height, weight, and waist circumference. Prior to multivariable logistic regression, we assessed multicollinearity among covariates using the variance inflation factor (VIF) and tolerance values [12]. All variables were evaluated within the same model. Based on these findings, we did not include all anthropometric variables simultaneously in the final adjusted models. Instead, we constructed separate multivariable models. The final reported odds ratios are derived from independent models, not a single saturated model including all anthropometric metrics simultaneously.

## 3. Results

### 3.1. General Characteristics

Mean age was 52.08 ± 17.11 y. The male–female was 15,190:19,182. The number of patients diagnosed with cataracts was 5909 (17.2%), and the number of patients with current cataracts was 2023 (5.9%). The number of patients diagnosed with AMD was 389 (1.1%), and the number of patients with current AMD was 273 (0.8%). The number of patients diagnosed with glaucoma was 743 (1.6%), and the number of patients with current glaucoma was 502 (1.5%). Overall data was described in Table 1.

### 3.2. Height and Ocular Diseases

Patients who had been diagnosed with cataracts had a shorter height (157.98 ± 9.05 cm) compared to those without a cataracts diagnosis (164.51 ± 9.10 cm; *p* < 0.001, *t*-test). Patients with current cataracts showed no difference in height. Patients who had been diagnosed with glaucoma had a shorter height (160.51 ± 9.38 cm) compared to those without a glaucoma diagnosis (163.55 ± 9.38 cm; *p* < 0.001, *t*-test). Patients with current glaucoma showed no difference in height. Patients who had been diagnosed with AMD had a shorter height (162.32 ± 8.76 cm) compared to those without AMD diagnosis (163.51 ± 9.39 cm; *p* = 0.014, *t*-test). Patients with current AMD showed no difference in height. When divided into two groups based on height, height was an associated factor for cataracts and glaucoma (*p* < 0.001 for both).

### 3.3. Weight and Ocular Diseases

Patients who had been diagnosed with cataracts had a lower weight (60.56 ± 10.72 Kg) compared to those without a cataracts diagnosis (65.32 ± 13.27 Kg; *p* < 0.001, *t*-test). Patients with current cataracts showed no difference in weight. Patients who had been diagnosed with glaucoma had a lower weight (62.26 ± 10.86 Kg) compared to those without a glaucoma diagnosis (64.63 ± 13.04 Kg; *p* < 0.001, *t*-test). Patients with current glaucoma showed no difference in weight. Patients who had been diagnosed with AMD showed no difference in weight. Patients with current AMD showed no difference in weight. When divided into two groups based on weight, weight was an associated factor for cataract and glaucoma (*p* < 0.001 for both).

### 3.4. Waist Circumference and Ocular Diseases

Patients who had been diagnosed with cataracts had a greater waist circumference (86.27 ± 9.49 cm) compared to those without a cataracts diagnosis (83.07 ± 10.74 cm; *p* < 0.001, *t*-test). Patients with current cataracts had a lesser waist circumference (86.04 ± 9.79 cm) compared to those without a cataracts diagnosis (86.62 ± 9.25 cm; *p* = 0.037, *t*-test). Patients who had been diagnosed with glaucoma had a greater waist circumference (86.24 ± 9.54 cm) compared to those without a glaucoma diagnosis (83.53 ± 10.63 cm; *p* < 0.001, *t*-test). Patients with current glaucoma showed no difference in waist circumference. Patients who had been diagnosed with AMD had a greater waist circumference (86.57 ± 10.08 cm) compared to those without an AMD diagnosis (83.56 ± 10.61 cm; *p* < 0.001, *t*-test). Patients with current AMD showed no difference in waist circumference. When divided into two groups based on waist circumference, a greater waist circumference was an associated factor for cataracts, glaucoma, and AMD (<0.001 for all).

### 3.5. BMI and Ocular Diseases

Patients who had been diagnosed with cataracts had a higher BMI (24.23 ± 3.27) compared to those without a cataracts diagnosis (24.02 ± 3.73; *p* < 0.001, *t*-test). Patients with current cataracts showed no difference in BMI. Patients who had been diagnosed with glaucoma showed no difference in BMI. Patients with current glaucoma showed no difference in BMI. Patients who had been diagnosed with AMD showed no difference in BMI. Patients with current AMD showed no difference in BMI.

### 3.6. Waist Circumference-to-Height Ratio and Ocular Diseases

Patients who had been diagnosed with cataracts had a higher waist circumference-to-height ratio (0.547 ± 0.061) compared to those without a cataracts diagnosis (0.505 ± 0.064; *p* < 0.001, *t*-test). Patients with current cataracts had a lower waist circumference-to-height ratio (0.545 ± 0.062) compared to those without a cataracts diagnosis (0.550 ± 0.060; *p* < 0.001, *t*-test). Patients who had been diagnosed with glaucoma had a greater waist circumference-to-height ratio (0.539 ± 0.63) compared to those without a glaucoma diagnosis (0.511 ± 0.65; *p* < 0.001, *t*-test). Patients with current glaucoma showed no difference in waist circumference-to-height ratio. Patients who had been diagnosed with AMD had a greater waist circumference-to-height ratio (0.533 ± 0.057) compared to those without an AMD diagnosis (0.512 ± 0.065; *p* < 0.001, *t*-test). Patients with current AMD showed no difference in waist circumference-to-height ratio. When divided into two groups based on waist circumference-to-height ratio, a greater waist circumference was an associated factor for cataracts, glaucoma, and AMD (*p* < 0.001 for all).

### 3.7. Associated Factor Analysis for Ocular Diseases

After adjusting for age, sex, height, weight, and waist circumference, the associated factors for cataracts were age (*p* < 0.001, OR 18.831, 95%CI 17.204–20.617), height (*p* < 0.001, OR 0.619, 95%CI 0.561–0.684), weight (*p* = 0.012, OR 0.777, 95%CI 0.638–0.946), and waist circumference (*p* < 0.001, OR 1.270, 95%CI 1.185–1.361), but not sex (*p* = 0.123, OR 1.077, 95%CI 0.980–1.184). The associated factors for glaucoma were age (*p* < 0.001, OR 4.677, 95%CI 3.907–5.598), weight (*p* = 0.050, OR 0.521, 95%CI 0.331–0.818), and waist circumference (*p* = 0.003, OR 1.277, 95%CI 1.089–1.498), but not height (OR 0.965, 95%CI 0.778–1.1971). The associated factor for AMD was age (*p* < 0.001, OR 4.348, 95%CI 3.410–5.544), but not sex, height, weight, and waist circumference.

**Figure 1:**
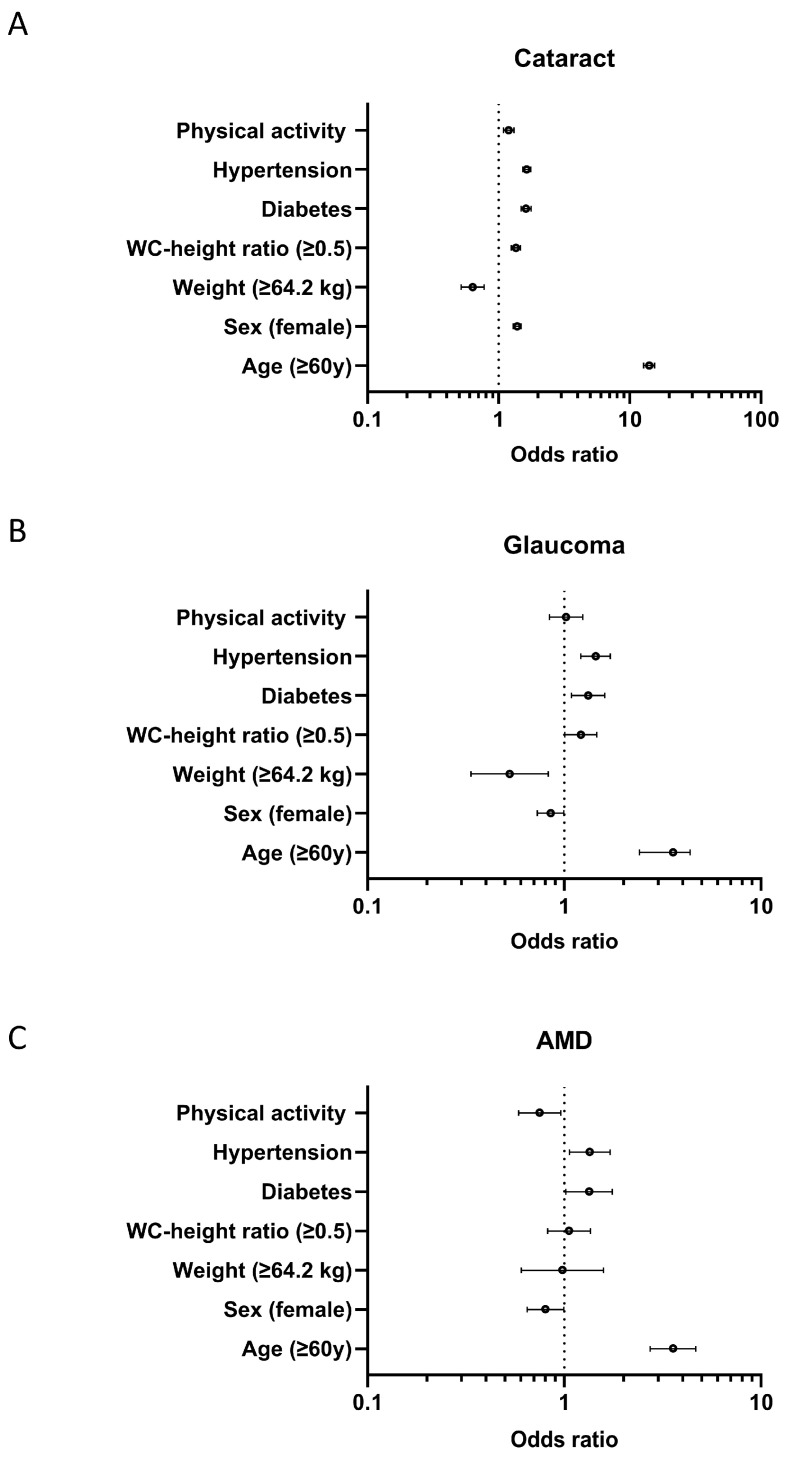
Forest plots showing the odds ratio with a 95% confidence interval. Forest plots display the estimated odds ratios (points) and 95% confidence intervals (horizontal lines) for key associated factors in relation to (**A**) cataracts, (**B**) glaucoma, and (**C**) age-related macular degeneration (AMD). Risk factors evaluated include physical activity, hypertension, diabetes, waist circumference-to-height ratio (WC-height ratio ≥ 0.5), weight (≥64.2 kg), female sex, and age ≥ 60 years. The vertical dotted line indicates an odds ratio of 1.0 (reference/no association). Odds ratios to the right of this line indicate increased risk, while those to the left indicate decreased risk for the corresponding eye disease.

After adjusting for age, sex, weight, waist circumference–height ratio, diabetes mellitus, hypertension, and physical activity, the associated factors for cataracts were age (*p* < 0.001, OR 14.041, 95%CI 12.765–15.444), sex (*p* < 0.001, OR 1.384, 95%CI 1.291–1.485), height (*p* < 0.001, OR 0.633, 95%CI 0.518–0.774), weight (*p* < 0.001, OR 0.633, 95%CI 0.518–0.774), waist circumference–height ratio (*p* < 0.001, OR 1.355, 95%CI 1.248–1.471), diabetes mellitus (*p* < 0.001, OR 1.614, 95%CI 1.479–1.762), hypertension (*p* < 0.001, OR 1.638, 95%CI 1.524–1.761), and physical activity (*p* < 0.001, OR 1.194, 95%CI 1.090–1.307) (Figure 1A). The associated factors for glaucoma were age (*p* < 0.001, OR 3.579, 95%CI 2.941–4.356), sex (*p* = 0.046, OR 0.853, 95%CI 0.731–0.997), weight (*p* = 0.006, OR 0.528, 95%CI 0.336–0.830), waist circumference–height ratio (*p* = 0.042, OR 1.214, 95%CI 1.007–1.463), diabetes mellitus (*p* = 0.005, OR 1.323, 95%CI 1.088–1.608), and hypertension (*p* < 0.001, OR 1.444, 95%CI 1.216–1.714), but not physical activity (OR 1.022, 95%CI 0.841–1.244) (Figure 1B). The associated factors for AMD were age (*p* < 0.001, OR 3.569, 95%CI 2.738–4.652), sex (*p* = 0.044, OR 0.803, 95%CI 0.648–0.995), diabetes mellitus (*p* = 0.036, OR 1.337, 95%CI 1.019–1.754), hypertension (*p* = 0.014, OR 1.347, 95%CI 1.063–1.708), and physical activity (*p* = 0.022, OR 0.750, 95%CI 0.587–0.959), but not weight (OR 0.979, 95%CI 0.605–1.584) and waist circumference–height ratio (OR 1.057, 95%CI 0.823–1.357) (Figure 1C).

### 3.8. Height and Refractive Error

Height was correlated with spherical error (r = −0.018, *p* = 0.005, Pearson correlation analysis), astigmatic error (r = 0.021, *p* < 0.001, Pearson correlation analysis) and BCVA (r = 0.171, *p* < 0.001, Pearson correlation analysis) of the right eye. Weight was correlated with spherical error (r = −0.024, *p* < 0.001, Pearson correlation analysis), and BCVA (r = 0.111, *p* < 0.001, Pearson correlation analysis) of the right eye. Waist circumference was correlated with astigmatic error (r = −0.042, *p* < 0.001, Pearson correlation analysis) and BCVA (r = −0.023, *p* < 0.001, Pearson correlation analysis) of the right eye. BMI was correlated with astigmatic error of the right eye (r = −0.0218, *p* < 0.001, Pearson correlation analysis).

## 4. Discussion

Our study presents comprehensive insights into the relationship between body metrics, such as height, weight, waist circumference, and BMI, and prevalent ocular diseases including cataracts, glaucoma, and AMD. The data suggest significant differences in body metrics between patients diagnosed with these ocular conditions and those without such diagnoses, highlighting potential physical health disparities linked to ocular health.

Firstly, we observed that patients diagnosed with cataracts, glaucoma, and AMD generally exhibited different physical characteristics when compared to those without these conditions. Notably, patients with diagnosed cataracts and glaucoma had shorter heights and lower weights than their counterparts, which aligns with previous studies suggesting that lower body size could be linked to the occurrence of these ocular conditions. In contrast, no significant differences in height or weight were found among patients with current manifestations of these diseases, indicating that the initial conditions might play a role in the development rather than the ongoing presence of the disease. Significant differences in height and weight were observed in those diagnosed with cataracts or glaucoma, but not in those currently affected. Body metrics change over time because of age-related weight loss or height reduction and post-diagnosis lifestyle changes. This study may have self-reported errors. Individuals categorized as “currently unaffected” may still have undetected or early-stage disease, especially in certain conditions, like glaucoma, which can be asymptomatic for long periods. In addition, longer survival with prior diagnosis might skew the anthropometric profile of that group. The absence of an effect may simply reflect survival bias. Although our dataset did not include biochemical measurements, we suggest IGF-1, oxidative stress, and systemic inflammation as putative biological mechanisms based on the prior literature. IGF-1, which regulates the height throughout childhood and adolescence. [13], has been implicated in the development of cataracts and AMD, and may also influence neuroinflammatory processes relevant to glaucoma [14,15,16]. Recent research into metabolic–vascular pathways could be integrated into the IGF-1-mediated mechanisms [17,18]. Emerging evidence suggests that microcirculatory dysfunction plays a key role in simultaneously affecting both retinal and central visual pathways. Metabolic syndrome induces microvascular alterations specifically in the retinal ganglion cell layer [18], while metabolic stress impairs neurovascular coupling in central visual pathways [17]. Interestingly, waist circumference was greater in patients diagnosed with cataracts, glaucoma, and AMD compared to those without such diagnoses. This supports the notion that central obesity could be an associated factor for the development of these ocular diseases. [4], possibly due to its association with systemic inflammation and vascular issues. [19], which are known associated factors for these conditions. Lower height and weight may reflect suboptimal nutritional status, potentially compromising the body’s antioxidative capacity [20]. Cataracts are known to be associated with oxidative damage to lens proteins [21]. In glaucoma and AMD, retinal cells and the optic nerve are particularly susceptible to oxidative insults [22,23]. Lower BMI may sometimes reflect chronic inflammation, sarcopenia, or biological frailty [24]. Such systemic conditions may contribute to increased ocular vulnerability through accelerated aging processes, including compromised cellular repair mechanisms in ocular tissues. Growth hormone, sex steroids, and thyroid hormones influence both somatic growth and the development of ocular diseases [25,26,27,28].

Furthermore, our study found no significant differences in BMI among patients with current cataracts, glaucoma, or AMD. This could suggest that BMI may play a role in disease development but not necessarily in current disease status [29,30]. While this study found no significant differences in BMI, other measures of obesity, such as waist circumference, showed significant differences. Thus, these measures may be better predictors of ocular disease risk than BMI [4,31,32]. BMI cannot differentiate between muscle mass and adipose tissue, potentially leading to misclassification of body composition [32].

Based on the statistical analysis of associated factors for cataracts, glaucoma, and AMD, distinct risk profiles emerge for these eye conditions, which could guide targeted interventions and screening strategies. For cataracts, the most significant associated factor is age, showing a very high odds ratio, implying that older individuals are substantially more likely to develop cataracts. This result is expected, as the lens of the eye naturally degrades with time [33]. Both sex and waist circumference are also significant, with females and individuals with a larger waist circumference being more prone to cataracts. Interestingly, height shows an inverse relationship with cataract risk, suggesting that taller individuals might have some protective factors against this condition. The association between height and cataracts has been controversial, with conflicting evidence from various studies [34]. Several study suggest a potential protective effect of greater height. [34,35], while some studies suggest that taller individuals may have a higher risk of cataracts [36]. The association may be influenced by other factors, such as diabetes, smoking, and family history of cardiovascular disease [37]. Waist circumference-to-height ratio, which is a useful measure for assessing overall obesity and abdominal fat [38], was an associated factor for cataracts. Diabetes, hypertension and physical activity are well-known associated factors for cataracts [39]. The associated factors of glaucoma also heavily involve age, though with a lower odds ratio compared to cataracts, indicating a less steep age-related increase in risk. The significant role of waist circumference aligns with prior research suggesting metabolic syndrome and related body fat distribution may influence intraocular pressure and blood flow to the optic nerve [40,41,42]. Notably, weight shows an inverse association with glaucoma risk, a finding that diverges from typical assumptions that higher body weight contributes to increased disease risk. Excessive body weight tends to lead to higher IOP, which means that high IOP becomes a major associated factor for glaucoma [7,43]. Diabetes, hypertension, and physical activity are the well-known associated factors for glaucoma [44]. For AMD, age is the only significant factor, underscoring its nature as a primarily age-related condition. The lack of association with sex, height, weight, and waist circumference suggests that these factors do not play a role in AMD development, suggesting the potential for different pathophysiological mechanisms from cataracts and glaucoma. These findings underscore the importance of age as a universal associated factor across all studied conditions but also show how body composition influences eye health differently depending on the specific eye disease. These data reinforce the need for personalized approaches in the prevention and management of ocular diseases, particularly in populations at higher risk due to physiological factors, like body size and composition. Diabetes and hypertension are also well-known associated factors for AMD [45]. Thus, early ophthalmologic screening is recommended not only for individuals with systemic diseases, such as diabetes and hypertension, but also for those with shorter stature, lower body weight, or a high waist circumference-to-height ratio. Further research into how these associated factors interact over time and under various environmental influences could refine these observations and improve preventive strategies.

Although the conventional cut-off for abdominal obesity in Asian men is a waist circumference of ≥90 cm, in this study, we used 84 cm as the threshold based on evidence from previous studies that identified this value as a more sensitive predictor of metabolic syndrome in Korean men [46,47].

Our analysis also showed significant correlations between body metrics and refractive errors, specifically astigmatism and spherical errors, as well as with BCVA. These correlations may underline the potential influence of body composition on eye health beyond direct ocular diseases, suggesting that overall physical health, including body weight and shape, could be integral to maintaining ocular health.

This study is a cross-sectional study, which has the limitations. One of the primary limitations is the inability to determine causality due to the simultaneous measurement of exposure and outcome [48]. To mitigate this, we employed statistical adjustments for potential confounders. However, further longitudinal or mechanistic studies are necessary. As the present analysis is based on data from the Korean National Health and Nutrition Examination Survey (KNHANES), information on disease severity and clinical classification was not available. Future studies incorporating detailed clinical data, including disease staging and severity indices, are necessary to better elucidate the relationship between anthropometric factors and ocular disease burden. KNHANES 2015–2021 did not include the measurement of body fat percentage, although body fat percentage may be a stronger predictor than other indices for health [49]. Future studies including body fat percentage may be helpful for assessing the association between body metrics and ocular diseases.

This study has other limitations that warrant consideration. First, we were unable to adjust for certain key confounders, most notably smoking status. Smoking is a well-established risk factor for major ocular diseases and is also associated with body composition changes. The lack of adjustment for smoking may, therefore, introduce residual confounding and could potentially bias our results [50,51]. Second, physical activity was assessed using a simple dichotomization rather than validated instruments, such as the International Physical Activity Questionnaire (IPAQ), which may reduce the precision and comparability of our findings [52]. Future studies should address these limitations by including more comprehensive confounder adjustment and standardized measurement of physical activity.

## 5. Conclusions

In conclusion, our findings advocate for a greater emphasis on monitoring physical health metrics as part of management and prevention strategies for ocular diseases. AMD was found to be more closely related to systemic diseases, such as diabetes and hypertension, than to body metrics. Further studies are needed to explore the causal relationships and mechanisms underlying these associations, which could lead to better targeted interventions aimed at reducing the risk of ocular diseases through the management of physical health.

## Figures and Tables

**Table 1:** Anthropometric and metabolic characteristics according to the diagnosis and current status of cataracts, glaucoma, and age-related macular degeneration (AMD).

			Height (cm)	Weight (Kg)	Waist Circumference (cm)	BMI (kg/m^2^)	Waist Circumference-to-Height Ratio
Cataracts	Diagnosed	No	164.51 ± 9.10	65.32 ± 13.27	83.07 ± 10.74	24.02 ± 3.73	0.505 ± 0.064
		Yes	157.98 ± 9.05	60.56 ± 10.72	86.27 ± 9.49	24.23 ± 3.27	0.547 ± 0.061
		*p*-value	<0.001 *	<0.001 *	<0.001 *	<0.001 *	
	Current	No	157.95 ± 9.25	60.55 ± 10.65	86.04 ± 9.79	24.26 ± 3.23	0.550 ± 0.060
		Yes	158.23 ± 8.70	60.89 ± 10.63	86.62 ± 9.25	24.27 ± 3.32	0.545 ± 0.062
		*p*-value	0.295	0.276	0.037 *	0.846	0.005 *
Glaucoma	Diagnosed	No	163.55 ± 9.38	64.63 ± 13.04	83.53 ± 10.63	24.06 ± 3.67	0.511 ± 0.65
		Yes	160.51 ± 9.38	62.26 ± 10.86	86.24 ± 9.54	24.11 ± 3.27	0.539 ± 0.63
		*p*-value	<0.001 *	<0.001 *	<0.001 *	0.676	<0.001 *
	Current	No	159.54 ± 8.90	61.51 ± 10.58	85.61 ± 7.41	24.18 ± 2.98	0.537 ± 0.053
		Yes	160.40 ± 9.55	62.01 ± 7.41	86.21 ± 9.90	24.03 ± 3.35	0.539 ± 0.066
		*p*-value	0.474	0.713	0.539	0.729	0.833
AMD	Diagnosed	No	163.51 ± 9.39	64.58 ± 13.01	83.56 ± 10.61	24.06 ± 3.67	0.512 ± 0.065
		Yes	162.32 ± 8.76	64.18 ± 11.78	86.57 ± 10.08	24.25 ± 3.35	0.533 ± 0.057
		*p*-value	0.014 *	0.544	<0.001 *	0.272	<0.001 *
	Current	No	163.45 ± 8.35	65.82 ± 12.86	86.83 ± 9.71	24.48 ± 3.47	0.532 ± 0.054
		Yes	162.52 ± 8.63	64.07 ± 11.55	86.65 ± 10.42	24.17 ± 3.41	0.533 ± 0.060
		*p*-value	0.496	0.347	0.911	0.571	0.895

* *p* < 0.05.

## Data Availability

All the data utilized in this study are publicly available through the KNHANES website Available online: https://knhanes.kdca.go.kr/knhanes/rawDataDwnld/rawDataDwnld.do (accessed on 14 July 2024).

## Abbreviations

The following abbreviations are used in this manuscript:

AMD	Age-related macular degeneration
BMI	Body mass index
CI	Confidence interval
OR	Odds ratio
